# The relationship between sunlight exposure duration and depressive symptoms: A cross-sectional study on elderly Chinese women

**DOI:** 10.1371/journal.pone.0254856

**Published:** 2021-07-16

**Authors:** Yufei Cui, Qiang Gong, Cong Huang, Feng Guo, Wang Li, Yongxiang Wang, Xin Cheng

**Affiliations:** 1 Institute of Exercise Epidemiology and Department of Physical Education, Huaiyin Institute of Technology, Huaian, Jiangsu Province, People’s Republic of China; 2 Department of Medicine and Science in Sports and Exercise, Tohoku University Graduate School of Medicine, Sendai, Japan; 3 Department of Sports and Exercise Science, College of Education, Zhejiang University, Hangzhou, Zhejiang Province, People’s Republic of China; Ehime University Graduate School of Medicine, JAPAN

## Abstract

Sunlight has been reported to have various beneficial effects on human health. Although research indicates an association between sunlight exposure and depressive symptoms, no study has examined it among the older adult population, especially among elderly Chinese women. This cross-sectional study addresses the aforesaid gap by investigating this association in 1,429 Chinese women aged 60 years and older. Information on their sunlight exposure was collected through a self-reported questionnaire. Depressive symptoms were assessed using the Zung Self-Rating Depression Scale (SDS). The logistic regression models revealed that greater exposure to sunlight is associated with a lower prevalence of depressive symptoms. In the final adjusted model, when the short sunlight exposure category (reference) was compared with the medium and long ones, the odds ratios and 95% confidence intervals for the prevalence of depressive symptoms (SDS cutoff ≥ 45) were 0.84 (0.60, 1.19) and 0.62 (0.43, 0.91), respectively (*p*-value for trend = 0.01). This significant association did not change when the SDS cutoff points were altered to ≥ 40 and ≥ 50. Findings indicate that an increased sunlight exposure duration is associated with a lower prevalence of depressive symptoms in elderly women.

## Introduction

Depression is among the most common psychological disorders that have a negative impact on peoples’ health and their quality of life [1]. In addition to the significant differences in its prevalence among races, cultures, and regions [2], depressive symptoms are also associated with many diseases, such as cardiovascular diseases (CVD), diabetes [3–5], and increased mortality [6]. Almost two-thirds of middle-aged and elderly people with depression suffer from CVD [7]. In recent decades, the rapid development of China’s economy resulted in an increase in income per capita, advancement in educational levels of people, and improvement in medical care services. Nevertheless, such progress has a downside for example, population urbanization leads to a significant increase in the number of elderly people living alone, convenient transportation causes a sharp drop in people’s physical activity, and people tend to suffer tremendous mental stress as a result of the increasing cost of living. All these factors have likely contributed to the growing prevalence of depression among the elderly in China. The country’s aging population and its proportion are expanding at a rapid rate; in 2017, 240.9 million people were aged over 60 years, accounting for 17.3% of the country’s total population [8]. Research has shown that a third of China‘s population aged over 75 years suffer from depression [9]. Women were found to be more likely to suffer from depression than men [10]. This finding warrants further research on ways to prevent and decrease depression among older adult women in China.

Sunlight exposure has long been considered to be a way to alleviate various diseases. Ultraviolet light is known to be used in treating several skin diseases, including atopic dermatitis, morphea, psoriasis, vitiligo, and mycosis fungoides [11]. The association between sunlight exposure and reduced cancer mortality in North America was identified in the 1960s [12]. Research suggests that sunlight also has beneficial psychological effects [13]. Peoples’ vitamin D levels are greatly affected by sunlight exposure [14]; older adults who have lower vitamin D serum levels were found to be more likely to have depressive symptoms [15]. This finding suggests that sunlight exposure may influence older adults’ depressive symptoms. Several studies have investigated this association [16, 17] but focused only on specific populations, such as middle-aged adults and postpartum women. To the researchers’ knowledge, there is no study that examines such an association in Chinese older adults. The differences between Chinese people and the population covered by previous studies in terms of, for example, living habits and cultural traditions necessitate further investigation of the former. Therefore, we designed a cross-sectional study to investigate the association between sunlight exposure and depressive symptoms among elderly Chinese women.

## Materials and methods

### Participants

This cross-sectional study was conducted from April to May 2019 at the health management center of the Jiuhua area, Fengxian District, Shanghai, China. Data were collected from a bone health examination of women aged 60 years and older. Participants joined the health examination voluntarily. An additional questionnaire survey was conducted after the examination. Written consent was obtained from the participants before they answered the survey. The study was approved by the Ethics Committee of the Huaiyin Institute of Technology. A total of 1,510 women agreed to participate in the study. Participants who were taking antidepressants, receiving psychological therapy, or younger than 60 years old were excluded (n = 42). Questionnaires with missing data were also excluded (n = 39). The final study population comprised 1,429 women.

### Assessment of sunlight exposure

Daily sunlight exposure was assessed by asking participants, “What is the average duration of your direct exposure to sunlight during each day?” [14] Response options were “<1 hour,” “1–2 hours,” “2–3 hours,” “3–4 hours,” and “> 4 hours.” Responses were consequently divided into three categories: “Short” (less than one hour of sunlight exposure), “Medium” (between 1–2 hours), and “Long” (more than two hours).

### Assessment of depressive symptoms

Depressive symptoms were measured using the Zung Self-Rating Depression Scale (SDS), a 20-item scale ranging from 20 to 80 points, that is used to assess the severity of depression. It is widely used in epidemiological studies to diagnose depressive symptoms [18, 19]. A high score indicates an increased severity of depressive symptoms. The reliability and validity of the SDS for the Chinese population have been previously demonstrated [20]. In the present study, depressive symptoms were considered to be present if the sum of the participants’ SDS scores was ≥ 45; cutoff values of 40 and 50 were used for sensitivity analysis.

### Confounding factors

Participants’ weight and height were also measured. Their body mass index (BMI) was calculated in kg/m^2^ and then divided into three categories: < 19 kg/m^2^, 19–24 kg/m^2^, and > 24 kg/m^2^. Information on participants’ age, former occupation, smoking and drinking status, household income, living conditions, educational levels, and physical activity levels were collected through the questionnaire. Participants were divided into white-collar and blue-collar workers, based on their former occupation. Their smoking status was categorized into smoker, former smoker, and non-smoker; and their drinking status into everyday drinker, occasional drinker, and non-drinker, based on their alcohol consumption. Annual household income was divided into three categories: ≤ CNY50,000 (low income), CNY50,001–CNY70,000 (middle income), and > CNY70,000 (high income). Educational level was divided into lower than high school and high school diploma or above. Physical activity was evaluated by the frequency of physical exercise, with more than six days per week being defined as “high physical activity,” one to five days per week as “middle physical activity,” and never exercising as “low physical activity.” Blood pressure was measured on the participants’ upper left arm with an automatic sphygmomanometer (KENTARO HBP-9021J, Japan). A second measurement was conducted if this value indicated hypertension (i.e., systolic blood pressure ≥ 140 mmHg, diastolic blood pressure ≥ 90 mmHg, or the use of an antihypertensive drug).

### Statistical analyses

A chi-squared test was used to evaluate proportions across the categories of sunlight exposure duration and covariates of interest. A logistic regression analysis was performed to examine the cross-sectional association between sunlight exposure duration and depressive symptoms. The results are presented as odds ratios (ORs) and 95% confidence intervals (CIs). The unadjusted (crude) associations were evaluated first, followed by subsequent adjustments for age and BMI (Model 1). Further adjustments were made for the participants’ educational level, former occupation, household income, living conditions, smoking and drinking habits, hypertension, diabetes, physical activity, and sleep quality (Model 2). A *p*-value of less than 0.05 was considered statistically significant for all analyses. The Statistical Product and Service Solutions version 24.0 for Windows (SPSS, Inc., Chicago, IL) was used in the analyses.

## Results

Participants’ characteristics according to the presence of depressive symptoms are presented in Table 1. Of the 1,429 participants, 352 (24.6%) had depressive symptoms. Participants with depressive symptoms were more likely to be older and have a higher BMI than those without. Women in blue-collar occupations with a low household income, or with a high educational level scored higher in the depression category. Non-smokers, non-drinkers, or participants with higher household incomes were more likely to have a lower incidence of depressive symptoms.

**Table 1:** Participants’ characteristics according to depressive symptoms.

	No depression	Depression	*p*-value^a^
	n = 1077	n = 352	
Age (n; %)			
60–69 years	683 (63.4)	164 (46.6)	< 0.001
70–79 years	271 (25.2)	129 (36.6)	
≥ 80 years	123 (11.4)	59 (16.8)	
BMI (n; %)			
< 19 kg/m^2^	31 (2.9)	13 (3.7)	0.018
19–24 kg/m^2^	471 (43.7)	124 (35.2)	
> 24 kg/m^2^	575 (53.4)	215 (61.1)	
Former occupation (n; %)			
White collar	208 (19.3)	76 (8.0)	< 0.001
Blue collar	869 (80.7)	324 (92.0)	
Smoking status (n; %)			
Smoker	8 (0.7)	14 (4.0)	< 0.001
Former smoker	9 (0.8)	0 (0.0)	
Non-smoker	1060 (98.4)	338 (96.0)	
Alcohol consumption (n; %)			
Everyday drinker	6 (0.6)	10 (2.8)	0.001
Occasional drinker	59 (5.5)	12 (3.4)	
Non-drinker	1012 (94.0)	330 (93.8)	
Household income in CNY (n; %)			
Low	359 (33.3)	157 (44.6)	< 0.001
Middle	349 (32.4)	88 (25.0)	
High	369 (34.3)	107 (30.4)	
Living alone (n; %)			
Yes	86 (8.0)	30 (8.5)	0.737
No	991 (92.0)	322 (91.5)	
Educational level (n; %)			
≥ High school	217 (20.1)	93 (26.4)	0.017
< High school	860 (79.9)	259 (73.6)	
Hypertension (n; %)			
Yes	63.4 (59.7)	195 (55.4)	0.170
No	434 (40.3)	157 (44.6)	
Diabetes (n; %)			
Yes	176 (16.3)	80 (22.7)	0.008
No	901 (83.7)	272 (77.3)	
Sleep quality (n; %)			
Poor	163 (15.1)	73 (20.7)	0.016
Normal	624 (57.9)	195 (55.4)	
Good	290 (26.9)	84 (23.9)	
Physical activity (n; %)			
Low	397 (36.9)	199 (56.5)	< 0.001
Middle	141 (13.1)	65 (18.5)	
High	539 (50.0)	88 (25.0)	

^a^Obtained using an *X*^2^ analysis for variables of proportion

Table 2 shows the association between sunlight exposure and depressive symptoms (SDS cutoff of 45) in elderly Chinese women. When compared with the short sunlight exposure category (reference), the ORs (95% CIs) of participants in the medium and long sunlight exposure categories were 0.84 (0.62, 1.14) and 0.68 (0.49, 0.95), respectively, in the crude model. In Model 1 (adjusted for age and BMI), the adjusted ORs (95% CIs) for depressive symptoms across categories of sunlight exposure were 1 for the short, 0.90 (0.65, 1.23) for the medium, and 0.67 (0.47, 0.94) for the long category. The same association was observed in Model 2 (final adjusted model).

**Table 2:** Adjusted associations between sunlight exposure and depressive symptoms among elderly women.

	Sunlight exposure			*p*-value for trend^a^
	Short	Medium	Long	
n	290	664	475	
Depressive symptoms (n)	83	167	102	
Crude model	1	0.84 (0.62, 1.14)^b^	0.68 (0.49, 0.95)^c^	0.024
Model 1^d^	1	0.90 (0.65, 1.23)	0.67 (0.47, 0.94)^c^	0.015
Model 2^e^	1	0.84 (0.60, 1.19)	0.62 (0.43, 0.91)^c^	0.011

^a^Obtained using a multivariate logistic regression analysis

^b^Results are expressed as odds ratios and 95% confidence intervals (for all such variables)

^c^Significantly different from the first category (*p* < 0.05)

^d^Adjusted for age and body mass index (BMI)

^e^Adjusted for age, BMI, educational level, former occupation, household income, living conditions, smoking and drinking habits, hypertension, diabetes, physical activity, and sleep quality

Table 3 presents the sensitivity analyses for the association between sunlight exposure and depressive symptoms (SDS cutoffs at 40 and 50 points) in elderly Chinese women. In the crude model, the ORs (95% CIs) relative to the short sunlight exposure group (reference) across all categories were measured: for the SDS cutoff ≥ 40, 0.78 (0.59, 1.02) for the medium and 0.66 (0.49, 0.89) for the long group; for SDS cutoff ≥ 50, 1.03 (0.66, 1.59) for the medium and 0.68 (0.41, 1.12) for the long group. The clear inverse associations did not change after being adjusted for confounding factors in both Models 1 and 2 for either of two SDS cutoff points.

**Table 3:** Sensitivity analyses for the associations between sunlight exposure and depressive symptoms among older women.

	Sunlight exposure			*p*-value for trend^a^
	Short	Medium	Long	
n	290	664	475	
Depressive symptoms (SDS ≥ 40)	140	279	181	
Crude model	1	0.78 (0.59, 1.02)^b^	0.66 (0.49, 0.89)^c^	0.006
Model 1^d^	1	0.80 (0.61, 1.07)	0.64 (0.47, 0.87)^c^	0.003
Model 2^e^	1	0.81 (0.60, 1.10)	0.61 (0.44, 0.86)^c^	0.003
Depressive symptoms (SDS ≥ 50)	32	75	37	
Crude	1	1.03 (0.66, 1.59)	0.68 (0.41, 1.12)	0.097
Model 1^d^	1	1.16 (0.74, 1.84)	0.68 (0.41, 1.13)	0.080
Model 2^e^	1	0.78 (0.47, 1.28)	0.50 (0.29, 0.87)^c^	0.010

^a^Obtained using a multivariate logistic regression analysis

^b^Results are expressed as odds ratios and 95% confidence intervals (for all such variables)

^c^Significantly different from the first category (p < 0.05)

^d^Adjusted for age and body mass index (BMI)

^e^Adjusted for age, BMI, educational level, former occupation, household income, living conditions, smoking and drinking habits, hypertension, diabetes, physical activity, and sleep quality.

## Discussion

In this population-based cross-sectional study, we investigated the association between sunlight exposure and depressive symptoms among elderly Chinese women. Results showed that a longer duration of sunlight exposure is significantly associated with a lower risk of depressive symptoms in this group. This association was not influenced by confounding factors such as age, BMI, or those related to health and lifestyle factors. Additionally, the association existed across different levels of SDS cutoffs.

Similar to our findings, a prior study indicated that higher levels of reported sunlight exposure are associated with fewer depressive symptoms in a middle-aged population in Australia [21]. Another cross-sectional study found that sunlight exposure is associated with a lower score in the Center for Epidemiology Studies Depression Scale (CES-D) in 1,907 Chinese university students [22]. A review article also reported that longer sunlight exposure is associated with a lower risk of depressive symptoms [23]. Although a direct comparison with our research is not appropriate because the participants and methods are different in these studies, all studies prove that sunlight exposure is associated with a lower risk of depression. Our findings reiterate those of previous studies and strengthen the evidence of the existence of this association. Our study is the first to suggest that sunlight exposure may also alleviate the depressive symptoms in elderly Chinese women. Therefore, exposure to sunlight may be an easy and safe way to prevent or alleviate depressive symptoms in this group.

The possible underlying mechanisms by which sunlight exposure alleviates depressive symptoms include several dimensions. First, ultraviolet radiation exposure is reported to increase the anti-inflammatory protein cytokine [24]. Inflammation is associated with depression [25]; thus, sunlight exposure may indirectly improve depressive symptoms through an increase in cytokine levels. Second, low levels of vitamin D are also associated with depressive symptoms. It is well known that exposure to sunlight produces vitamin D [26]. Third, sunlight helps to decrease the levels of serum cortisol and assists in the regulation of the cortisol circadian rhythm [27]. Cortisol is a stress hormone that also induces depression. Fourth, exposure to sunlight is known to lead to more positive mood [23], which consequently improves depressive symptoms. People who have high physical activity levels tend to have longer durations of sunlight exposure, which in turn helps to alleviate depressive symptoms. Although we considered physical activity as a confounding factor or mediator of this association, after adjusting for it, the significant inverse association between the duration of sunlight exposure and depressive symptoms did not change. This proved that in this study, physical activity effects cannot be attributable to the association between these two variables.

Questionnaire surveys are usually used in psychiatric studies to assess the severity of depressive symptoms. Common questionnaires include the SDS, CES-D, and Patient Health Questionnaire. However, the definition of depressive symptoms (i.e., a scale’s cutoff point) in previous studies was different. For example, the evaluation criteria of depressive symptoms using the SDS was usually defined at cutoff points of 40, ≥ 45, and 50, whereas the CES-D would defined them at cutoff points of 16 and 7 [18, 19, 28–30]. Considering that depressive symptoms have different definitions in each scale, it is necessary to perform a sensitivity analysis for these types of studies. In our study, in addition to an SDS cutoff point of 45, we also used 40 and 50 for the sensitivity analysis. Therefore, our results may be more reliable for measuring the association between sunlight exposure and depressive symptoms.

However, there are several limitations to our study. The first is because of the nature of cross-sectional studies, which does not allow for inferring the direction of causality. Second, a self-reported questionnaire was used for evaluating sunlight exposure; thus, a recall bias may exist. Third, the exclusion of participants with missing data may have resulted in some bias in our study sample. Fourth, participants in our study were from one area in Shanghai, China. Therefore, they may not be representative of populations in other areas. Finally, although we collected data on basic confounding factors, we cannot exclude the possibility that depressive symptoms are affected by other factors associated with sunlight exposure, such as food and nutrition intake.

In conclusion, the present study found that greater sunlight exposure is associated with lower levels of depressive symptoms among elderly Chinese women. The results of our study confirm the previous findings on the association between these two factors and provide important evidence for women’s mental health and implications for the field of preventive medicine and health education. The clinical significance and causal nature of the reported associations require further study.
